# Effects of whole-body vibration on cognitive function: a systematic review and meta-analysis

**DOI:** 10.1007/s11357-025-01914-0

**Published:** 2025-09-30

**Authors:** Ji-Woo Seok, Jaeuk U. Kim, Jung-Dae Kim

**Affiliations:** 1 Digital Health Research Division, Korea Institute of Oriental Medicine, 1672, Yuseong-Daero, Yuseong-Gu, 34054 Daejeon, South Korea; 2 KM Convergence Science, University of Science and Technology, Daejeon, South Korea

**Keywords:** Whole-body vibration, Cognitive function, Meta-analysis, Global cognition, Cognitive domains, Dose

## Abstract

Whole-body vibration exercise (WBV) is a low-intensity intervention for improving cognitive function, particularly among individuals who are older or have physical limitations. However, quantitative evidence regarding its effectiveness is limited. This meta-analysis aimed to quantify the effects of WBV on cognitive function and explore major moderating variables and differences across cognitive domains. Sixteen randomized controlled trials (RCTs) were included, yielding 37 effect sizes (Hedges' g) related to cognitive outcomes. Effect sizes were estimated for overall cognitive function and four subdomains: global cognition, executive function, attention, and memory. A meta-regression analysis was conducted to assess the influence of potential moderators, including vibration frequency, intensity, posture, and intervention duration. WBV had a small but statistically significant positive effect on overall cognitive function (Hedges' g = 0.375, 95% CI = [0.263, 0.486], p < .001). Effect sizes were greatest for global cognition (g = 0.55), followed by executive function (g = 0.41), attention (g = 0.28), and memory (g = 0.28). Meta-regression analysis revealed that most of the moderating variables were not significant. The total intervention dose was the only significant moderator (p = .041). This meta-analysis revealed that WBV is associated with meaningful improvements in cognitive function and that the benefits may be greater among populations with cognitive impairment. WBV may be clinically applicable as a low-intensity, accessible cognitive intervention for older adults and individuals with physical limitations. Future research should investigate the relationships between cognitive domain-specific responses and physiological indicators and develop tailored intervention strategies based on age and cognitive status.

**Registration:** PROSPERO CRD420251067063.

**Supplementary Information:**

The online version contains supplementary material available at 10.1007/s11357-025-01914-0.

## Introduction

Cognitive function is a higher-order mental process that selectively attends to external stimuli, encodes and retains information, and leads to appropriate behavior through judgment and executive control [1]. Cognitive function consists of various subdomains, such as memory, attention, executive function, language, visuospatial processing, and reasoning; cognitive impairment occurs if one or more of these subdomains are impaired [2].

Cognitive impairment is caused by various pathophysiological mechanisms, such as age-related physiological changes, neurodegenerative diseases, cerebrovascular disorders, trauma, and metabolic conditions [3–6], and in severe cases, it can even affect activities of daily living. According to a recent study, the risk of death in elderly individuals with cognitive impairment is 1.76 times greater than that in elderly individuals without cognitive impairment [7], suggesting that cognitive decline is directly related to survival rates beyond simple functional deterioration.

Cognitive decline is common not only in aging individuals but also in individuals with neurodevelopmental disorders such as attention-deficit/hyperactivity disorder (ADHD) and chronic physical conditions such as diabetes. ADHD patients experience difficulties in academic and occupational functioning because of impairments in complex cognitive domains such as executive function and working memory [8], and individuals with diabetes may experience cognitive decline because of metabolic abnormalities such as insulin resistance or deficiency, which negatively affects disease self-management and prognosis [9]. Therefore, early intervention and prevention of cognitive decline due to various causes are emerging as important clinical challenges [10–13].

However, to date, pharmacological treatments for cognitive decline have shown limited efficacy and present challenges related to side effects and treatment adherence [14]. Accordingly, interest in nonpharmacological interventions such as cognitive training, psychosocial interventions, and physical activity is increasing. Among these, physical activity-based interventions can induce changes in brain structure and function related to cognitive improvement and have the advantages of safety, accessibility, and facilitation of mind‒body interactions [15, 16].

Whole-body vibration exercise (WBV) is a novel approach that can stimulate various sensory and motor systems simultaneously with relatively low intensity, in addition to conventional aerobic or resistance exercise [17]. WBV is a physical activity intervention that stimulates neuromuscular reflexes and induces systemic physiological responses by delivering low-frequency mechanical vibrations to the body through a vibration platform [18]. WBV has been reported to have beneficial effects on musculoskeletal function, balance, cardiovascular function, and body composition [19–23]. WBV may also have the potential to improve cognitive function [17, 24–26].

Recent systematic reviews have focused on the potential of WBV as an intervention method that can improve cognitive function. Shantakumari and Ahmed [25] reported that WBV may enhance cognitive function in mice by increasing neuroplasticity and synaptic density in the hippocampus [25]. Wen, Leng [26] conducted a systematic review of 18 studies on the relationship between WBV and cognitive function and suggested that WBV may have measurable cognitive benefits not only in patients with cognitive impairment but also in healthy adults [26]. Halmai, Holsgrove [24] examined the acute effects of WBV on various functions, such as visual perception, postural stability, and motor control, and reported that the results for cognitive function were inconsistent because of differences in the sensitivity of the assessment tools or experimental design [24]. Moreover, Yang [27] reported that the effect of WBV varies depending on conditions such as vibration frequency, intensity, and intervention duration and that low-frequency and short-term interventions may have positive effects on cognitive function in particular [17]. In summary, these findings suggest that WBV is an intervention capable of inducing neurophysiological stimulation with relatively low physical demand and may serve as a safe and effective approach to improve cognitive function, especially among older adults or individuals with limited mobility.

Nevertheless, previous WBV studies on cognitive function have several limitations. First, most studies are based on small sample sizes and heterogeneous research designs, which substantially limits the interpretability of the findings. Second, few studies have systematically compared outcomes across different WBV parameters, such as frequency, amplitude, posture, and intervention intensity, which makes determining the optimal intervention conditions difficult [17]. Third, analyses that distinguish effects by cognitive subdomain are lacking, which hinders the identification of which specific domains are most responsive. Fourth, existing systematic reviews have either included only a subset of studies or lack meta-analytic approaches, making it difficult to compare effects quantitatively. For example, Shantakumari and Ahmed [25] included only adult populations and conducted descriptive analyses, whereas Wen, Leng [26] examined individuals with neurological conditions but did not analyze vibration characteristics or domain-specific cognitive outcomes [25, 26].

Therefore, this study aimed to systematically and quantitatively synthesize the effects of WBV on cognitive function. To this end, we conducted a comprehensive review of randomized controlled trials (RCTs) and randomized crossover studies across diverse age groups and clinical populations. The primary outcome was overall cognitive function, defined as the pooled meta-analytic estimate across all reported cognitive domains. Meta-regression analyses were additionally performed to examine whether vibration parameters (e.g., amplitude, frequency, posture, and total intervention dose) moderated the effects. Subgroup analyses were conducted to explore domain-specific outcomes, including attention, memory, executive function, and global cognition. Finally, the overall certainty of evidence was appraised using the Grading of Recommendations, Assessment, Development, and Evaluation (GRADE) framework, and the implications for future research and clinical application were discussed.

## Methods

### Study design

This study used a meta-analysis, meta-regression analysis, and systematic review methodology to empirically demonstrate how whole-body vibration interventions affect cognitive function across age groups. This systematic review and meta-analysis was conducted in accordance with the Preferred Reporting Items for Systematic Reviews and Meta-Analyses (PRISMA) 2020 guidelines [28].

The review protocol was prospectively registered on the International Prospective Register of Systematic Reviews (PROSPERO; registration number CRD420251067063). Amendments were made to the registered protocol after registration. Specifically, the age range of the eligible population was broadened to include children as well as older adults, and the cognitive outcome categories were simplified (e.g., episodic and working memory combined; processing speed included under attention). In addition, only WBV interventions and randomized controlled/crossover trials were analyzed to enhance the methodological rigor. These amendments were introduced to improve feasibility and validity and are fully reflected in the Methods and Results section.

### Literature search and screening

We derived key concepts by utilizing both medical subject headings (MeSH) and free-text keywords. The search strategy was organized around three concepts: Concept 1 (cognitive function OR cognition OR global cognition OR memory OR attention OR executive function), Concept 2 (whole-body vibration OR vibration OR WBV), and Concept 3 (randomized controlled trial OR clinical trial OR crossover trial OR crossover study OR crossover design). The literature search was conducted in six databases (PubMed, Embase, PsycINFO, Web of Science, Scopus, and the Cochrane Central Register of Controlled Trials (CENTRAL) via the Cochrane Library). No restrictions were applied regarding the publication date. The search language was limited to English.

More specific search strategies for each database are presented in Tables S1–S6. The literature published up to May 2024 was searched, and additional relevant studies were explored by checking the reference lists of the identified studies. All the literature derived from the search results was imported into EndNote 20 (Clarivate Analytics, London, UK) to remove duplicate literature. The literature published up to May 2024 was searched, and additional relevant studies were explored by checking the reference lists of the identified studies. All the literature derived from the search results was uploaded to EndNote 20 (Clarivate Analytics, London, UK) to remove duplicate literature. After the title and abstract were reviewed, potentially relevant literature was initially selected, and the full text was checked to determine whether to include it. The literature selection process was conducted independently by two researchers, and if there was disagreement, a third researcher made the final decision through mediation. The literature selection process was conducted independently by two researchers, and if there was disagreement, a third researcher made the final decision through mediation.

### Eligibility criteria

This systematic review and meta-analysis were conducted in accordance with PICOS principles. The study subjects were limited to studies that evaluated the effects of vibration therapy on cognitive function regardless of age, sex, or race. The subjects included both the general population without a history of neurological or psychiatric disorders and people with clinical conditions that may affect cognitive function, such as dementia, attention-deficit hyperactivity disorder (ADHD), and spinal musculoskeletal disorders (e.g., lumbar lordosis). Studies with mixed populations were also included if the patients’ cognitive function outcomes were reported separately by group. Animal studies and studies on occupational or transportation-related vibration exposure were excluded. Interventions included WBV, but only studies that provided structured vibration through a mechanical device for the purpose of improving cognitive function were included. Vibration was allowed in various positions, such as standing, sitting, and squatting, and the physical parameters of the vibration, such as frequency (Hz), amplitude (mm), and duration, had to be clearly reported. Studies that aimed solely at improving physical function or that used only manual vibration, manual stimulation, or auditory or electromagnetic vibration without mechanical delivery were excluded.

The controls included no intervention, sham vibration conditions (e.g., standing on a platform without vibration), general exercise or cognitive training, or pre-post comparisons within a crossover design. Studies in which WBV was combined with another intervention and the control group received the same intervention alone (e.g., experimental group = WBV + exercise, control group = exercise) were included. This allows for estimation of the pure effect of WBV. On the other hand, studies in which WBV was combined with an additional intervention but the control group received no treatment or sham vibration (placebo) were excluded, as it becomes difficult to separate the effects of WBV from those of the additional intervention. The study designs included randomized controlled trials (RCTs), nonrandomized controlled trials (nRCTs), and crossover designs and had to provide pre- and postintervention comparison data on cognitive function. Observational studies, single-group pre- and posttreatment comparative studies, case reports, qualitative studies, meta-analyses, introductory papers, conference abstracts, and non-peer-reviewed literature were excluded.

### Data extraction

The following information was systematically extracted from the final selected studies: researcher name and publication year, study design type (randomized controlled study, nonrandomized controlled study, crossover study, etc.), number of participants and average age, clinical characteristics (e.g., diagnosis), intervention conditions (posture during vibration application, time of one intervention, number of trials per week, and total intervention period), vibration platform settings (frequency, amplitude, gravitational acceleration, etc.), intervention type of the control group (e.g., no intervention, sham vibration, general exercise, etc.), and cognitive function assessment tools and measured cognitive domains (e.g., global cognition, memory, attention, executive function, etc.). In particular, the data were organized on the basis of the ‘WBV Big Five’, including key variables related to vibration intervention, such as frequency, amplitude, application method (i.e., platform type or posture), time and frequency per session, and total intervention period [29]. When figures were provided in the form of images rather than tables, data were extracted using WebPlotDigitizer, and when statistics such as the mean and standard deviation were not specified, additional data were requested from the corresponding author via email. Data extraction was performed independently by two researchers, and in cases of disagreement, the final decision was made through mediation by a third researcher.

### Assessment of methodological quality

The methodological quality of each included study was independently assessed by two assessors using the standard version of the Cochrane Risk of Bias 2 (RoB 2) tool [30]. For studies using a crossover design, a specialized version of RoB 2 for crossover studies was applied. The RoB 2 tool assesses the risk of bias in five domains: (1) bias in the randomization process, (2) bias due to deviations from intended interventions, (3) bias due to missing outcome data, (4) bias in outcome measurement, and (5) bias in the selection of reported outcomes. In addition, for crossover design studies, bias arising from period and carryover effects (Domain S) was also assessed to account for the potential confounding of intervention effects across the two periods [31]. Each item was rated as ‘low risk of bias’, ‘some concerns’, or ‘high risk of bias’ according to the RoB 2 guidelines. In cases where the opinions of the two evaluators did not match, an agreement was reached through discussion with a third evaluator.

### Statistical analysis

In this meta-analysis, Hedges’ g was used as the effect size metric, as it provides a small-sample correction to Cohen’s d and offers more accurate estimates in studies with limited sample sizes [32]. Effect sizes were calculated with 95% confidence intervals and weighted using the inverse variance method. A pre-post correlation coefficient of r = 0.5 was assumed where necessary [33, 34].

All the statistical analyses, including the random-effects meta-analysis, were conducted using JASP (v0.19.0.0) to account for methodological and clinical heterogeneity across studies [34–36]. The restricted maximum likelihood (REML) method was used to estimate the effect size [37], and heterogeneity was assessed using Cochran's Q statistic, τ^2^ (variance estimate), I^2^ (heterogeneity ratio), and H^2^ (heterogeneity coefficient) [30, 38].

To assess the robustness of the results, potential publication bias was examined using both visual and statistical methods. A funnel plot was first generated to visually inspect asymmetry in the distribution of effect sizes [39]. For quantitative assessment, Kendall’s τ rank correlation and Egger’s regression tests were conducted [40, 41]. In addition, Orwin’s fail-safe N was calculated to estimate how many missing studies would be needed to nullify the observed effects, thereby evaluating the stability of the findings [42].

The influence of each study on the overall analysis was assessed using influence diagnostics, including standardized residuals, difference in fitted values (DFFITS), Cook's distance, and hat values. Analysis and visualization were performed using forest plots, funnel plots, and diagnostic plots [43].

In this study, meta-regression analysis was conducted to explain the heterogeneity of the overall effect size [44]. The meta-regression model was estimated as a random-effects model using the REML method, and the moderating variables included vibration parameters (e.g., amplitude type, frequency type, vibration type, posture type), population characteristics (e.g., cognition type, age group, participant type), intervention and study characteristics (e.g., intervention type, control type, outcome domain, study design), and total intervention dose [45]. Each moderator was included in the regression equation as a categorical or continuous variable, and the Wald test and omnibus test of model coefficients were applied to evaluate the reduction in effect size heterogeneity and statistical significance [45].

In addition to the overall effect on cognitive function, subgroup analysis was performed to explore the effects of vibration interventions on specific cognitive domains more specifically. The analysis was conducted on the basis of the cognitive domains reported in each study and categorized into the following four subdomains: (1) attention, (2) executive function, (3) memory, and (4) global cognition. Each subgroup analysis was performed using the REML method under a random effects model, and heterogeneity indices (I^2^, τ^2^) and publication bias tests were also conducted along with effect size estimation [45].

### Certainty of evidence

The certainty of evidence was assessed using the Grading of Recommendation, Assessment, Development, and Evaluation (GRADE) approach, which considers five domains: risk of bias, inconsistency, indirectness, imprecision, and publication bias. On the basis of these criteria, evidence was rated as very low, low, moderate, or high [46]. Two authors independently conducted the assessments and resolved any disagreements through discussion.

## Results

### Study selection

**Fig. 1:**
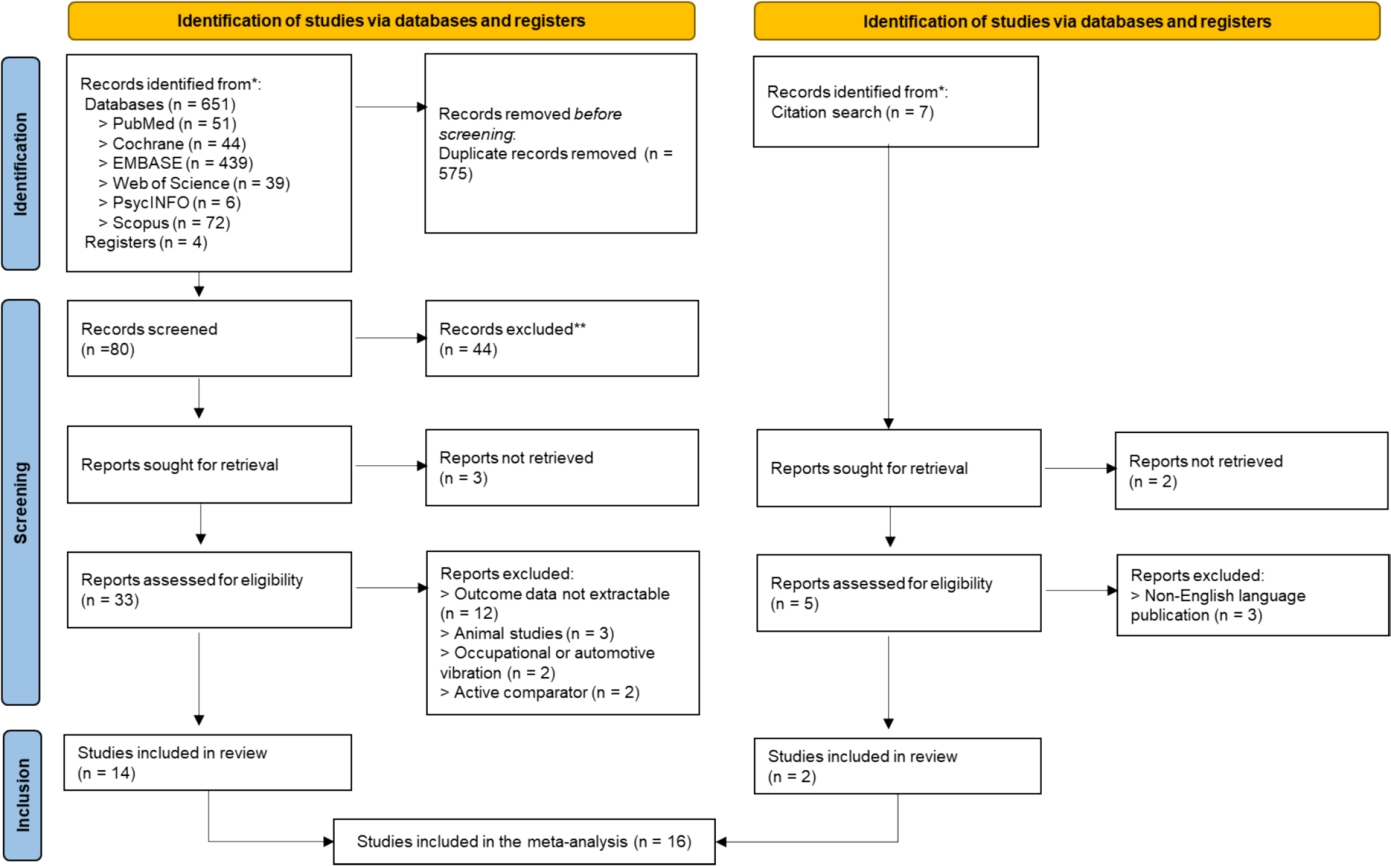
PRISMA flow chart for the study selection process

A total of 655 articles were identified as a result of the literature search, and 80 of them were included in the screening review after removing duplicates. Forty-four articles were excluded through title and abstract review, and 33 articles were subjected to full-text review. Among these, 19 articles were excluded for reasons such as unavailable outcome data, animal studies, studies related to work/vehicle vibration, unavailability of full text, comparisons of non-WBV interventions, and non-English articles. A total of 16 studies were ultimately included in the meta-analysis (Fig. 1 and Tables S1–S6).

### Characteristics of the studies

The characteristics of the 16 studies included in this meta-analysis are summarized in Table 1. The publication years ranged from 2015 to 2025. The participants covered a wide age spectrum, from children with ADHD (mean age ≈ 8 years) to healthy older adults in their 80 s and 90 s. Studies in healthy populations included young adults (18–28 years) and middle-aged to older adults (42–99 years). These studies investigated mainly short-term WBV effects on executive function, memory, and attention, with outcomes assessed by tools such as the ImPACT, Stroop, TMT, and DSST. Studies in clinical populations have targeted a variety of groups. Older adults with mild dementia (≈80 years) and advanced dementia (≈86 years) showed improvements in global cognition and executive function. In those at risk of falling (≈75 years), WBV combined with psychomotor training enhanced executive and cognitive–motor performance. Adults with dynapenia (< 65 years) and patients with multiple sclerosis (≈54 years) demonstrated improvements in global cognition and attention/executive function, respectively. Improvements were also reported in children with ADHD (≈8 years) and in young adults with chronic stroke (≈22 years).

**Table 1:** Study characteristics of the 16 studies selected for the meta-analysis

Author & year	Type of study	Participants	Sample (N, Age)	Intervention & Control Protocol	Intervention Protocol	WBV Parameters	Posture & WBV Type	Device Type	Outcome Measures	Adherence & Adverse Events
*Healthy populations*										
Amonette et al. (2015)	Randomized crossover study	Healthy adults	I1: 12 (28.2 ± 6.4), I2: 12 (28.2 ± 6.4), C: 12 (28.2 ± 6.4)	I1: vertical WBV; I2: rotational WBV C: no vibration	Freq: 1/week, Dur: 1 wk	Bouts: 5, Bout Dur: 2 min, Rest: 1 min between bouts, Freq: 30 Hz, Amp: 4 mm	Static hip-width stance squats, with the knees positioned at a 45 degrees angle of flexion. (vertical, rotational)	Power Plate vibration platform (Power Plate USA), Galileo 2000 vibration platform	ImPACT (Verbal Memory, Visual Memory)	100% completion; no adverse events except itching of feet
Boerema et al. (2018)	Randomized controlled trials	Healthy adults	I: 18 (42–99), C: 16 (45–90)	I: WBV C: sham	Freq: 4/week, Dur: 5 wk	Bouts: 1, Bout Dur: 4 min, Rest: None, Freq: 30 Hz, Amp: 0.5–1 mm	Seated on a chair mounted on vibration platform (vertical)	Pactive Motion (Rolstoelpod)	Stroop -CW; TMT-B; DS (Forward, Backward)	NR; NR
Faes et al. (2020)	Randomized controlled trials	Healthy adults	I: 35 (23.97 ± 4.21), C: 35 (22.26 ± 2.80)	I: WBV C: sham	Freq: 1/week, Dur: 1 wk	Bouts: 5, Bout Dur: 1 min, Rest: 1, Freq: 6 Hz, Amp: 3 mm	Standing upright with slightly bent knees (Side-alternating)	SRT-Zeptor Medical plus Noise (FreiSwiss AG, Zurich, Switzerland)	Stroop-CW	100% completion; NR
Hamer et al. (2025)	Randomized crossover experiment	Healthy adults	I: 24 (21.50 ± 1.59), C: 24 (21.50 ± 1.59)	I: WBV C: sham	Freq: 1/week, Dur: 1 wk	Freq: 12 and 24 Hz	Sitting or standing during WBV (Side-alternating)		Stroop-CWI; CBT	100% completion; NR
Yang et al. (2023)	Randomized controlled trials	Healthy older adults	I: 22 (72.7 ± 5.6), C: 20 (71.0 ± 4.9)	I: WBV C: usual routin	Freq: 3/week, Dur: 8 wk	Bouts: 5, Bout Dur: 1 min, Rest: 1, Freq: 20 Hz, Amp: 2 mm	Standing barefoot, knees bent 20°, trunk upright, hands on handlebars (Side-alternating)	Galileo Med-L (Germany)	MMSE	Adherence 95.65%; no major adverse events except mild itching (n = 3)
de Bruin et al. (2020)	Randomized controlled trials	Healthy older adults	I: 9 (86.1 ± 5.9), C: 8 (90.3 ± 5.9)	I: WBV C: sham	Freq: 3/week, Dur: 8 wk	Bouts: 5, Bout Dur: 1 min, Rest: 1 min between bouts, Freq: 3–6 Hz, Amp: 3 mm	Standing barefoot with slight flexion of ankle/knee/hip (Side-alternating)	Zeptor med® plus Noise (Frey AG, Zurich, Switzerland)	TMT-A; TMT-B	100% completion; no adverse events
He et al. (2025)	Randomized controlled trials	Healthy adults	I: 32 (18–26), C: 32 (18–26)	I: WBV C: no vibration	Freq: 7/week, Dur: 2 wk	Bouts: 6, Bout Dur: 2 min, Rest: 2 min between bouts, Freq: 30 Hz, Amp: 0–0.5 mm	Standing with slight knee flexion, holding handrails (Vertical)	Power Plate my5 (My5R Vibration Technology)	DSST	NR; NR
Tsai et al. (2024)	Randomized controlled trials	Healthy older adults	I: 22 (68.09 ± 4.26), C: 22 (66.05 ± 6.64)	I: WBV + BFR C: Blood Flow Restriction (BFR) only	Freq: 1/week, Dur: 1 wk	Bouts: 10, Bout Dur: 1 min, Rest: 1, Freq: 13 Hz, Amp: 3 mm	Isometric squat (static knee flexion at 100°) (vertical)	TP1 (Turtle Gym, Changhua, Taiwan)	MDMT-S	NR; NR
*Clinical populations*										
Durgut et al. (2020)	Randomized controlled trials	Children with ADHD	I: 15 (8.33 ± 1.17), C: 15 (7.93 ± 1.22)	I: Treadmill training + WBV C: Treadmill training	Freq: 3/week, Dur: 8 wk	Bouts: 1, Bout Dur: 15 min, Rest: 5, Freq: 50 Hz, Amp: 0–5 mm	Standing without hand support (vertical)	Crazy FIT-N vibration platform	Stroop-TBAG; BRIEF	100% completion; no adverse events
Kim et al. (2018)	Randomized controlled trials	Older adults with mild dementia	I: 9 (79.22 ± 4.02), C: 9 (81.44 ± 3.75)	I: WBV C: no vibration	Freq: 5/week, Dur: 8 wk	Bouts: 5, Bout Dur: 2 min, Rest: 1 min between bouts, Freq: 20–35 Hz	Standing squatting and sumo squat position (vertical)	Whole body vibration exerciser (VM-10, Korea)	MMSE	90% completion; NR
Heesterbeek et al. (2019)	Randomized controlled trials	Older adults with dementia	I: 30 (86.2 ± 4.7), C: 30 (85.8 ± 7.4)	I: WBV C: usual routine	Freq: 4/week, Dur: 6 wk	Bout Dur: 4 min, Freq: 30 Hz, Amp: 1–2 mm	Seated (Side-alternating WBV)	Pactive Motion	MMSE; Stroop (Word, Color, Interference); DS (Forward, Backward); VF (Phonemic, Semantic)	93.33% completion; NR
Rosado et al. (2021)	Randomized controlled trials	Older adults at risk of falling	I: 16 (74.7 ± 5.5), C: 19 (76.8 ± 5.8)	I: psychomotor training + WBV C: daily activities	Freq: 3/week, Dur: 24 wk	Bouts: 4–6, Bout Dur: 0.75–1 min, Rest: 60 s, Freq: 12.6–15 Hz, Amp: 3 mm	Standing with bent knees (Side-alternating)	Galileo®Med35	CogTUG	88.89% completion; NR
Rosado et al. (2022)	Randomized controlled trials	Older adults at risk of falling	I: 16 (74.7 ± 5.5), C: 16 (75.9 ± 5.7)	I: psychomotor training + WBV C: psychomotor training	Freq: 3/week, Dur: 24 wk	Bouts: 4–6, Bout Dur: 0.75–1 min, Rest: 60 s, Freq: 12.6–15 Hz, Amp: 3	Semiflexed knee stance (~ 30° knee flexion) (Side-alternating)	Galileo® Med35 (Novotec Medical, Germany)	TMT-A; TMT-B	88.89% completion; NR
Su et al. (2024)	Randomized controlled trials	Older adults with dynapenia	I: 29 (< 65), C: 29 (< 65)	I: WBV C: usual routine	Freq: 3/week, Dur: 12 wk	Bouts: 10, Bout Dur: 1 min, Rest: 30 s between bouts, Freq: 25–40 Hz, Amp: 2.5–5 mm	Standing (exact posture not specified) (vertical)	BW760B (Taiwan)	MMSE	100% completion; no adverse events
Yang et al. (2022)	Randomized controlled trials	Patients with Multiple Sclerosis	I: 9 (53.9 ± 4.9), C: 9 (48.8 ± 11.7)	I: WBV C: usual routine	Freq: 3/week, Dur: 6 wk	Bouts: 5, Bout Dur: 1–1.5 min, Rest: 1, Freq: 20 Hz, Amp: 2 and 4 mm	Standing barefoot, knees flexed at 20°, trunk upright, holding handlebar (Side-alternating)	Galileo (Novotec Medical GmbH)	PASAT-3; SRT; BRIEF-A (Metacognition Index, Global Executive Composite)	Adherence 100%; no major adverse events except itching of legs (n = 1)
Yule et al. (2015)	Randomized crossover trial	Patients with chronic stroke	I: 6 (21.7 ± 1.96), C: 6 (21.7 ± 1.96)	I: WBV C: no vibration	Freq: 3/week, Dur: 4 wk	Bouts: 5–7, Bout Dur: 1 min, Rest: 1 min between bouts, Freq: 22–26 Hz, Amp: 2.1–6.5 mm	Standing static squat (~ 110° knee flexion) (Side-alternating)	Galileo Sport (Novotec Medical GmbH, Germany)	ACE-III	NR; NR

Abbreviations: *ACE-III* Addenbrooke’s Cognitive Examination-III, *ADHD* Attention Deficit Hyperactivity Disorder, *Amp* Amplitude, *BFR* Blood Flow Restriction, Bouts, Vibration bouts (sessions within a treatment), *BRIEF* Behavior Rating Inventory of Executive Function, *BRIEF-A* Behavior Rating Inventory of Executive Function – Adult Version, *CBT* Color Block Test, *C* Control group, *CogTUG* Cognitive Timed Up and Go, *DS* Digit Span, *DS-F* Digit Span Forward, *DS-B* Digit Span Backward, *DSST* Digit Symbol Substitution Test, *Dur* Duration, *Freq* Frequency, *GEC* Global Executive Composite, *I* Intervention group, *ImPACT* Immediate Post-Concussion Assessment and Cognitive Testing, *MCI* Mild Cognitive Impairment, *MDMT-S* Modified Delayed-Matching-to-Sample Task, *MI* Metacognition Index, *MMSE* Mini-Mental State Examination, *NR* Not reported, *PASAT-3* Paced Auditory Serial Addition Test (3-s version), *SRT* Selective

Across all the studies, most used randomized controlled designs, with intervention frequencies ranging from once to seven times per week and durations ranging from 1 to 24 weeks. The vibration frequencies ranged from 3 to 50 Hz, the amplitudes ranged from 0.5 to 6.5 mm, and the postures included standing, semisquatting, and seated positions. Side-alternating platforms were most commonly applied, followed by vertical devices. Cognitive outcomes were measured using standardized tools for global cognition (MMSE, ACE-III), executive function (Stroop, TMT, BRIEF), attention (PASAT-3, DSST), and memory (Digit Span, SRT, ImPACT). Adherence was generally high across studies, often exceeding 90%, and no serious adverse events related to WBV were reported; only mild, transient symptoms such as itching were occasionally noted (Table 1).

### Quality assessment results

The Revised Cochrane Risk of Bias Tool (RoB 2.0) was used to assess the risk of bias of the 16 studies included in this meta-analysis. Three of these studies used a crossover design, for which the crossover-specific version of RoB 2.0 was applied. In terms of the randomization process, a total of eight studies (50%) were assessed as having some concerns, as they lacked sufficient information on random sequence generation or allocation concealment methods. In the area of deviations from intended interventions, 9 studies (56.3%) were assessed as low risk, as the interventions were implemented as planned and the possibility that the practitioners or participants could influence the results was low. In contrast, six studies (37.5%) were assessed as high risk, as the practitioners were not blinded or the intervention conditions were not properly followed, and appropriate analyses (e.g., ITT analysis) were therefore not conducted.

In the missing outcome data domain, 14 studies (87.5%) were assessed as low risk, indicating that there was little missing data or that an appropriate missing data handling method was applied. Two studies (12.5%) were classified as high risk because of high dropout rates or insufficient explanations for missing data handling.

In the measurement of the outcome domain, 12 studies (75%) were assessed as low risk since, in most cases, the assessors were blinded to group allocation, or the outcome measures were objective. In contrast, three studies (18.75%) were assessed as high risk because the assessment was not blinded or involved multiple assessors without consistency procedures. In the selection of the reported results domain, seven studies (43.75%) were assessed as low risk, and six studies (37.5%) were assessed as having some concerns because of the absence of a prespecified analysis plan. Three studies (18.75%) were assessed as high risk, as they may have selectively reported statistically significant outcomes.

Overall, only 2 studies (12.5%) were rated as low risk across all domains, 3 studies (18.75%) were rated as having some concerns in one or more domains but not high-risk domains, and 10 studies (68.75%) were rated as high risk in one or more domains and thus were classified as having a high overall risk of bias. Both crossover studies were rated as having a high overall risk because of concerns regarding randomization procedures, potential carry-over effects, and selective outcome reporting (Fig. S1).

### Effect size of WBV on cognition

**Fig. 2:**
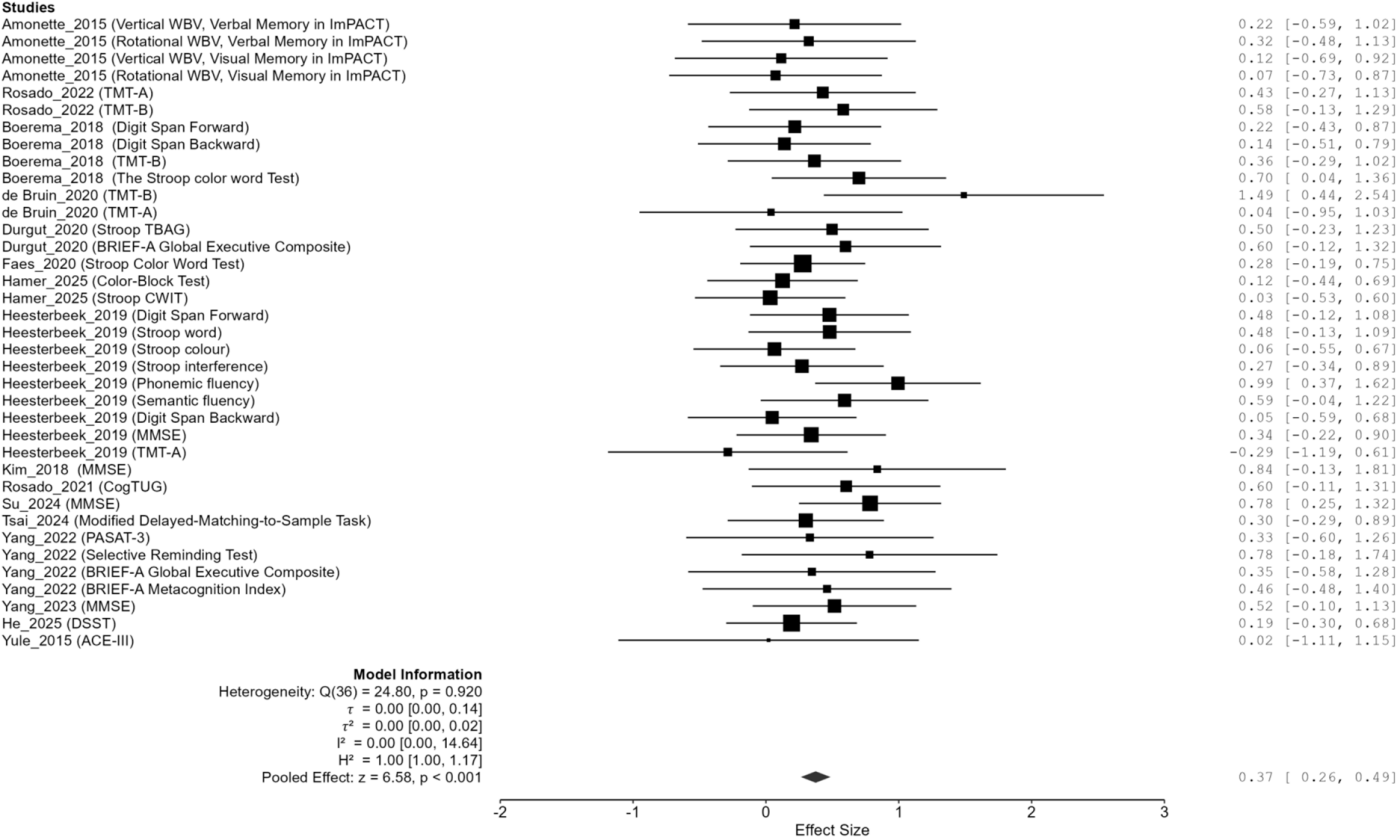
Meta-analysis of the effects of WBV on cognition

A meta-analysis of the effects of whole-body vibration (WBV) interventions on cognitive function revealed that the overall effect size estimate was 0.375 (standard error = 0.057), which was statistically significant (z = 6.58, p < 0.001; 95% confidence interval: 0.263–0.486) (Fig. 2). These findings suggest that the interventions included in the analysis have a small but statistically significant positive effect on improving cognitive function. The heterogeneity analysis revealed that the Cochran's Q statistic was 24.80 (df = 36, p = 0.920), τ^2^ = 0.000, I^2^ = 0.0% (0.0–14.6%), and H^2^ = 1.000, indicating that there was little difference in the effect size between the included studies. These results indicate that the model fit of this meta-analysis is good and that the heterogeneity is very low.

Egger's regression asymmetry test, which assesses publication bias, also revealed a nonsignificant result (p = 0.518), suggesting that the asymmetry of the funnel plot is low and that the possibility of publication bias is limited. In addition, Rosenthal's fail-safe N value was calculated to be 551, indicating that at least 551 missing studies would be needed to invalidate the statistically significant overall effect, which implies that the likelihood of the observed effect being due to chance is extremely low (Fig. S2 and Table S7).

The results of the study-by-study impact analysis revealed that most studies did not have an excessive influence on the overall analysis results. The greatest positive values in terms of standardized residuals were observed for de Bruin_2020 (TMT-B) (2.085) and Heesterbeek_2019 (phonemic fluency) (standardized residual = 1.980), whereas the greatest negative values were observed for Heesterbeek_2019 (TMT-A) (− 1.449) and Hamer_2025 (Stroop CWIT) (− 1.209) (Table S8).

### Meta-regression analysis of the moderating variables affecting the cognitive effects of WBV

In this study, the overall heterogeneity was low, and there was little difference in the effect size between the included studies. Nevertheless, to explore the influence of various moderating variables on the effect size on the basis of a priori hypotheses, a meta-regression analysis was performed on a total of 12 moderating variables. As a result, none of the moderating variables showed statistically significant effects, except for the total dose.

**Fig. 3:**
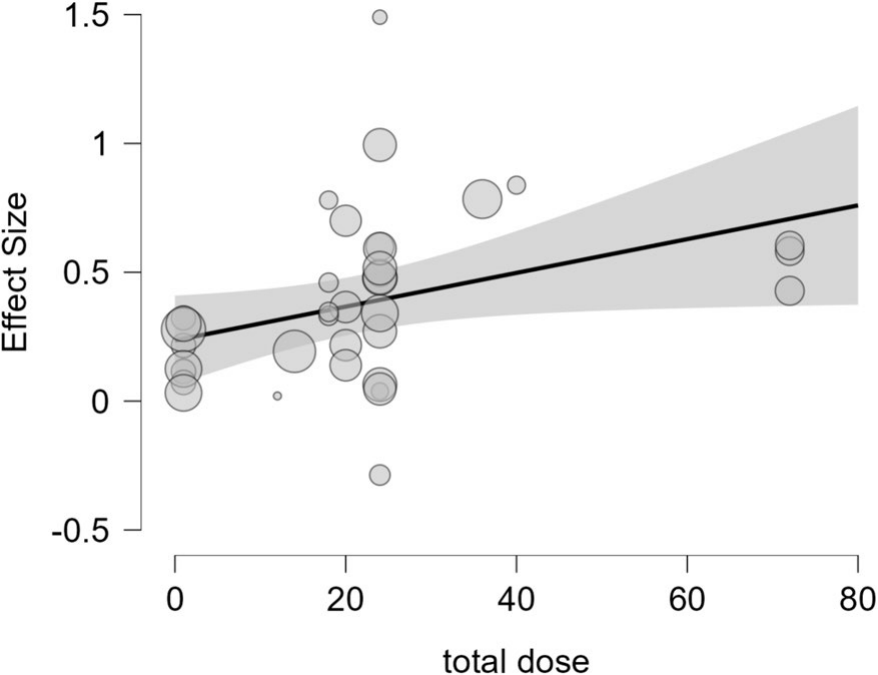
Meta-regression of total WBV dose and cognitive function effect size

In the meta-regression analysis including the total dose as a continuous variable, the overall model was statistically significant (Q = 4.180, df = 1, p = 0.041). The regression coefficient was 0.007 (standard error = 0.003), indicating that the greater the total dose, the greater the improvement in cognitive function tended to be. This relationship can be confirmed through the scatter plot and regression line presented in Fig. 3.

In contrast, no significant moderating effect was found in the models including the remaining 11 moderating variables. The results of the omnibus tests for these models were as follows: for amplitude type, Q = 0.506, df = 2, p = 0.776; for frequency type, Q = 3.570, df = 2, p = 0.168; for vibration type, Q = 0.128, df = 1, p = 0.720; for posture type, Q = 0.086, df = 1, p = 0.770; for cognition type, Q = 0.060, df = 1, p = 0.806; for age type, Q = 3.33, df = 2, p = 0.189; for intervention type, Q = 0.014, df = 1, p = 0.907; for participant type, Q = 5.81, df = 6, p = 0.445; for control type, Q = 0.200, df = 1, p = 0.655; and for study design, Q = 3.844, df = 1, p = 0.050. The model including outcome type was also not statistically significant (Q = 3.499, df = 3, p = 0.321). Therefore, in this meta-regression analysis, only the total intervention dose was identified as a variable that significantly explained the variation in effect size related to cognitive function (Table 2).

**Table 2:** Results of the meta-regression analyses for the moderator variables of WBV effects on cognitive function

Moderator Variable	Level	Reference Level	β	95% CI	*z*	*p* value	Omnibus Q(df)	Omnibus *p*
Peak Amplitude type	2–3 mm	1 mm	0.104	[− 0.213, 0.421]	0.644	0.519	0.506 (2)	0.776
	4–5 mm		0.116	[− 0.231, 0.463]	0.657	0.511		
Frequency type	10–29 Hz	< 10 Hz	− 0.077	[− 0.490, 0.336]	− 0.365	0.715	3.570 (2)	0.168
	30–50 Hz		0.275	[− 0.249, 0.799]	1.029	0.303		
Vibration type	Side-alternating	Vertical	− 0.043	[− 0.277, 0.191]	− 0.358	0.720	0.128 (1)	0.720
Posture type	Seated	Standing	− 0.034	[− 0.265, 0.196]	− 0.293	0.770	0.086 (1)	0.770
Cognition type	Cognitively impaired	Cognitively healthy	0.029	[− 0.203, 0.261]	0.246	0.806	0.060 (1)	0.806
Age type	Youth	Adults	0.322	[− 0.226, 0.871]	1.152	0.249	3.33 (2)	0.189
	Older adults		0.209	[− 0.033, 0.451]	1.692	0.091		
Intervention type	Combined WBV	WBV alone	0.016	[− 0.247, 0.278]	0.117	0.907	0.014 (1)	0.907
Participant type	ADHD	Healthy population	0.288	[−0.251, 0.826]	1.047	0.295	5.81 (6)	0.445
	Chronic stroke		−0.241	[−1.385, 0.902]	−0.414	0.679		
	Dementia		0.128	[−0.137, 0.394]	0.949	0.343		
	Dynapenia		0.523	[−0.038, 1.083]	1.826	0.068		
	Multiple Sclerosis		0.228	[−0.181, 0.638]	1.092	0.275		
	Risk of falling		0.276	[−0.165, 0.716]	1.225	0.221		
Control type	Active	Passive	− 0.069	[− 0.373, 0.234]	− 0.447	0.655	0.200 (1)	0.655
Outcome domain	Executive function	Memory	0.195	[−0.089, 0.478]	1.347	0.178	3.499 (3)	0.321
	Global cognition		0.324	[−0.058, 0.705]	1.661	0.097		
	Attention		0.055	[−0.337, 0.448]	0.276	0.782		
Study design	Randomized controlled trials	Randomized crossover study	0.301	[1.091*10^–4^, 0.602]	1.961	0.050	3.844 (1)	0.050
Total dose	-	Continuous	0.007	[2.708*10^–4^, 0.013]	2.045	0.041*	4.180 (1)	0.041*

*CI* Confidence interval, *WBV* Whole-body vibration, *RCT* Randomized controlled trial

For each categorical moderator, the reference level is shown in the second column. The regression coefficient (β) indicates the difference in effect size compared with the reference level. The omnibus Q and *p* values refer to the significance of the moderator variable as a whole. The total dose variable (frequency per week × duration in weeks) was analyzed as a continuous predictor

*p* <.05 is considered to indicate statistical significance and is indicated with an asterisk (*)

### Subgroup analysis of WBV effects across cognitive domains

**Fig. 4:**
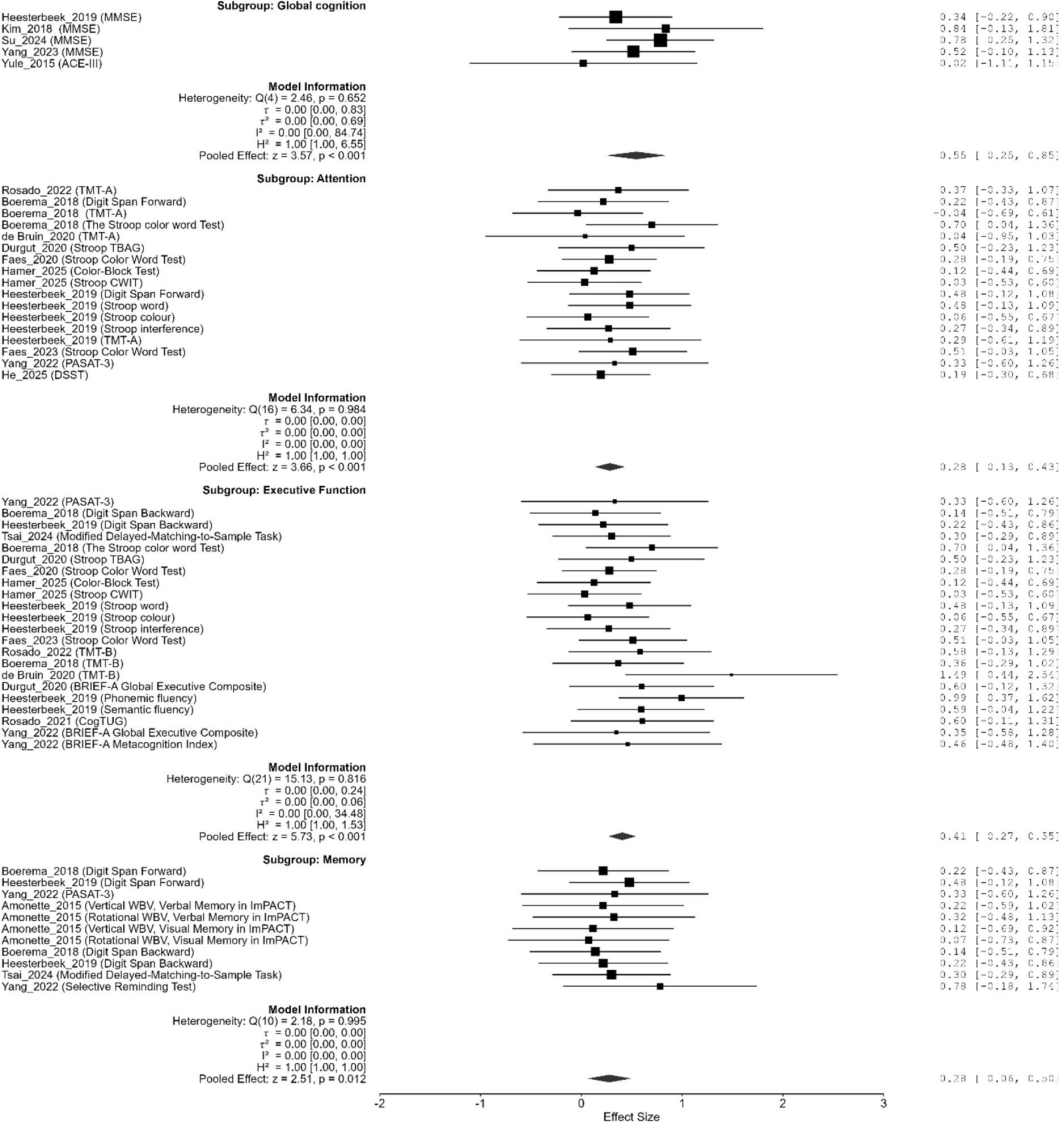
Forest plots showing the effects of WBV on four cognitive domains. Effect sizes (Hedges’ g) and 95% confidence intervals are displayed for each study across the following domains: global cognition, attention, executive function, and memory. Pooled estimates were calculated using a random-effects model

To determine whether the effect of WBV intervention on improving cognitive function differed across cognitive domains, subgroup analysis was conducted on attention, executive function, global cognition, and memory. As a result, WBV intervention had statistically significant effects on all four cognitive domains (Fig. 4). In the executive function domain, Hedges' g = 0.41 (95% CI = [0.27, 0.55]), z = 5.73, p < 0.001, I^2^ = 0.0%. This can be interpreted as a small-to-moderate effect. In the global cognition domain, Hedges' g = 0.55 (95% CI = [0.25, 0.85]), z = 3.57, p < 0.001, I^2^ = 0.0%, and the pooled effect was the greatest among the four domains. In the attention domain, the combined effect size was Hedges' g = 0.28 (95% CI = [0.13, 0.44]), z = 3.66, p < 0.001, I^2^ = 0.0%. In the memory domain, a significant improvement was observed, with Hedges' g = 0.28 (95% CI = [0.06, 0.50]), z = 2.51, p = 0.012, I^2^ = 0.0%, which is interpreted as a small effect (Fig. 4).

Residual heterogeneity tests were not statistically significant in any cognitive domain (all p ≥ 0.65), indicating that the robustness of the findings was not meaningfully affected. In addition, Rosenthal's fail-safe N (global cognition: 16; memory: 15; executive function: 264; attention: 66) exceeded the conventional threshold in all subdomains, suggesting a low risk of publication bias (Fig. S3). Subgroup differences across domains were not significant (Qₘ(3) = 3.43, p = 0.330).

### GRADE evaluation of the certainty of evidence for WBV-induced cognitive improvements

Table S9 presents the results of the GRADE assessment. The evidence for the improvement in overall cognitive function was assessed as moderate, as most studies had a low risk of bias or some concerns regarding risk of bias, whereas a few were at high risk owing to selective reporting and missing outcome data. The evidence for memory and attention in the subcognitive domains was rated as moderate. While several included studies presented some risk of bias, no significant concerns were noted in other assessment domains, such as inconsistency, indirectness, or imprecision. The evidence for executive function was rated low, mainly because of a high risk of bias in numerous studies and significant methodological flaws, including poor randomization and the absence of blinding in the outcome assessment. The evidence for global cognition was rated very low because of the limited number of studies, many of which had a high risk of bias, and the broad 95% confidence interval for the effect size. The evidence for the impact of WBV intervention on cognitive function is rated as moderate to very low, necessitating consideration of potential differences across subcognitive domains.

## Discussion

### Therapeutic effects of WBV on cognitive function

The results of this meta-analysis revealed that WBV had a small but statistically significant positive ability to improve overall cognitive function (Hedges' g = 0.375, SE = 0.057, p < 0.001, 95% CI = [0.263, 0.486]). The observed effect exhibited minimal heterogeneity (I^2^ = 0.0%), indicating strong homogeneity across the included studies. The robustness of the findings was further supported by various indicators, including Egger’s regression test for publication bias and the fail-safe N value (N = 551).

In the subgroup analysis by cognitive domain, statistically significant effects were observed across all the domains. Notably, the greatest effect size was found in the global cognition domain (Hedges' g = 0.55, 95% CI = [0.25, 0.85]), followed by executive function (g = 0.41), attention (g = 0.28), and memory (g = 0.28). These effects are interpreted as small to moderate effects, and although heterogeneity was minimal across all domains (I^2^ = 0.0%), the residual heterogeneity tests were not statistically significant, supporting the stability of the results. Although the overall and domain-specific effect sizes were statistically significant, it remains important to consider whether these changes translate into clinically meaningful improvements. For instance, an effect size of g = 0.41 in executive function may represent a modest gain, but whether this results in functional benefits in daily life tasks remains unclear. Future trials should integrate functional outcome measures or anchor-based analyses to assess real-world impact.

These findings are partially consistent with those of previous studies [25, 26]. A systematic review revealed improvements in executive function, attention, memory, and global cognitive function across various populations, including children with ADHD, older adults with cognitive impairment, and healthy individuals, with a particular emphasis on greater effectiveness in elderly individuals with dementia [25]. Similarly, the largest effect size in the present study was observed in the global cognition domain, which is likely attributable to the use of measures such as the MMSE, which is typically administered to populations with mild cognitive impairment or dementia. These findings suggest that the cognitive-enhancing effects of WBV may be more pronounced in individuals with preexisting cognitive deficits. Additionally, Wen, Leng [26] conducted a meta-analysis focusing on MMSE and Stroop test outcomes. The reported mean difference (MD) in the MMSE score was 0.52 points (95% CI: −0.26 to 1.3), suggesting a modest improvement in global cognition. These findings are comparable to the results observed in the present study (Hedges' g = 0.55, 95% CI = [0.25, 0.85]), further reinforcing the conclusion that WBV interventions are associated with positive changes in global cognitive function [26].

In contrast, Wen et al.’s meta-analysis yielded inconsistent findings for the Stroop test. The Stroop color-block test showed an MD of −0.06 (95% CI: −0.400.28), and the Stroop color-word test showed an MD of −2.07 (95% CI: −9.064.93), both indicating negligible or nonsignificant effects [26]. However, the present study analyzed executive function using a broader range of cognitive assessments, including but not limited to the Stroop task, and revealed a statistically significant effect (Hedges' g = 0.41, 95% CI = [0.27, 0.55]). This discrepancy is likely due to the inclusion of more comprehensive executive function measures, such as the Trail Making Test-B and Digit Span Backward, which may be more sensitive for detecting changes induced by WBV interventions. Furthermore, differences in analytical approaches may account for some of the variation in findings. Wen, Leng [26]. compared postintervention scores between the intervention and control groups, whereas the present study calculated change scores (Δ values) from pre- to postintervention and then compared these changes between the groups. This method allows for better control of individual baseline differences, resulting in more precise and sensitive estimates of intervention effects.

Moreover, some of the included studies reported that WBV had minimal or no effect on cognition. For example, Santin-Medeiros et al. [47] reported no improvement in cognitive function in elderly women following eight months of WBV training [47]. Similarly, Lam et al. [48] reported no significant effect of WBV on MMSE scores in individuals with mild to moderate dementia [48]. These null findings may be attributable to variations in participant characteristics, vibration frequency or intensity, body posture during WBV, or the sensitivity of the cognitive measures employed.

In conclusion, the results of this meta-analysis indicate that WBV is a promising therapeutic modality for enhancing cognitive function. While the effect sizes observed are generally small to moderate, the overall direction of the evidence favors a beneficial impact of WBV, particularly in populations with cognitive impairments. The most pronounced improvements were observed in the global cognition domain, suggesting the potential clinical relevance of WBV as a low-intensity, accessible intervention for older adults or individuals with physical limitations. These findings contribute to the growing body of literature supporting the utility of WBV in cognitive health and provide a foundation for its expanded application in clinical settings.

### Key factors modulating WBV’s therapeutic effects

As a result of this meta-regression analysis, most of the included moderating variables did not explain significant differences in the effect size, but only the total dose of intervention had a significant moderating effect (Q = 4.180, df = 1, p = 0.041). The regression coefficient was 0.007 (SE = 0.003), indicating that the greater the dose of intervention was, the greater the effect size on improving cognitive function. These findings suggest that WBV intervention affects cognitive function through the interaction of the cumulative intensity and duration of vibration rather than through a single factor.

On the other hand, Yang et al. [49] reported that the cognitive effects of WBV depend primarily on frequency, amplitude, and exposure time. They reported that short-term low-frequency vibration is likely to positively affect cognitive function (especially learning and memory), whereas prolonged high-intensity exposure may induce chronic stress on neural tissue, potentially leading to cognitive decline [17]. For example, in a preclinical study, memory decline and brain damage were observed in rats when 30 Hz WBV was applied for 4 h a day for 2–8 weeks [50]. In addition, another study suggested that frequent and long-term exposure to WBV is associated with the risk of back pain, sciatica, and spinal degenerative changes [51]. These prior findings are somewhat inconsistent with the present results, which may be attributable to contextual factors such as differences in study design and subject characteristics. Yang et al. [49] relied primarily on individual clinical and preclinical studies without conducting a meta-analytic synthesis [17]. In contrast, the present study analyzed quantitative effects using data from 15 RCTs, yielding a total of 37 effect sizes across different cognitive outcomes, which included various populations and vibration parameters. Second, as shown in Table 1, the majority of WBV protocols in this study were generally aligned with the safe and effective parameters proposed by Yang [27] and Halmai, Holsgrove [24], thereby minimizing the likelihood of adverse effects [17, 24]. Consistent with this, our review revealed no reports of serious adverse events across the included trials, supporting the overall safety of WBV when it is delivered in short bouts of 10–15 min. Moreover, while previous reports have highlighted the potential negative effects of WBV on cognitive function, such findings were predominantly observed in animal models subjected to long-term vibration exposure exceeding four hours per day [50]. In contrast, several human studies have reported that when vibration exposure is limited to 15–30 min, short-term improvements in memory and attention are observed [26]. Finally, although some studies targeting older adults have reported that excessively long WBV sessions may reduce attentional focus, the current study included participants across a wide age range and various clinical populations, with WBV session durations mostly restricted to 10–15 min, thereby minimizing the risk of cognitive fatigue.

Additional evidence supporting a dose–response relationship is also available. For example, a meta-analysis of stroke patients revealed that WBV significantly improved balance and mobility, with these effects significantly correlated with the total intervention dose (r = 0.649–0.785, p < 0.05) [52]. They calculated the intervention dose as the product of vibration intensity and exposure time, which, similar to the results of the present study, suggests that the cumulative stimulation dose represents a key predictor of therapeutic efficacy.

In summary, these findings indicate that intervention dose functions as a key moderating variable in the cognitive benefits of WBV. The direction and magnitude of the moderating effect appear to depend on factors such as intervention design, measurement outcomes, and participant characteristics. The present study empirically demonstrated that total stimulation dose is a more robust moderator of cognitive improvement than vibration frequency or exposure time is, distinguishing it from prior studies through its comprehensive meta-analytic scope encompassing diverse WBV protocols. Future research should aim to determine optimal vibration parameters (frequency, amplitude, and duration) tailored to the characteristics of specific populations and cognitive domains while concurrently establishing safety guidelines to prevent potential adverse effects from prolonged WBV exposure.

### Neurophysiological mechanisms underlying the effect of WBV on cognitive function

The results of this study suggest that WBV may have a positive effect on cognitive function and that this effect can be explained by various physiological and neurological mechanisms. Although the mechanism of cognitive enhancement by WBV has not yet been fully elucidated, several physiological hypotheses have been proposed [17, 26]. WBV activates peripheral mechanoreceptors, especially Meissner corpuscles, which are sensitive to approximately 30 Hz vibration [17, 53]. The sensory input induced by this process is transmitted to the primary somatosensory cortex via the spinothalamic tract and posterior medial spinal pathway [17, 53, 54]. This signal is subsequently transmitted to the prefrontal cortex, which is critically involved in working memory and executive function [55–57], and can influence information processing related to learning and memory through indirect connections with limbic structures such as the amygdala and hippocampus [58, 59].

In particular, EEG-based research has reported that sensory stimulation from WBV is associated with increased neural transmission efficiency and synaptic connectivity in the prefrontal cortex and induces an increase in alpha wave activity, thereby promoting overall cortical arousal [60]. In addition, a study by Herrero, Menendez [61] revealed that heart rate changes after WBV were closely correlated with activity in the prefrontal cortex as well as with the sympathetic/parasympathetic balance [61]. Changes in heart rate and oxygen consumption following WBV are thus associated with activation of the prefrontal cortex and regulation of the autonomic nervous system, and EEG analysis confirmed increased alpha wave activity in the prefrontal region, contributing to cortical activation [60, 61].

In addition, WBV is known to significantly affect the forebrain region through the activation of the cholinergic system [62]. In animal experiments, a rapid increase in the expression of the C-fos gene was observed in the cerebral cortex region, which is involved in motor-sensory processing, learning, and memory in individuals receiving WBV [57, 63]. C-fos plays important roles in enhancing neurotransmission and inducing the expression of genes involved in the production of proteins related to neuroplasticity and long-term memory formation [57, 62]. In addition, an elevated level of arousal following vibration intervention has been reported [64], suggesting that WBV may positively influence overall cognitive function and the arousal state of the brain beyond simple sensory stimulation.

In support of these physiological and molecular biological mechanisms, several preclinical studies have also reported the effects of WBV on enhancing cognitive function. For example, Keijser, Van Heuvelen [65] reported significant improvements in attention and motor performance after 5 weeks of WBV training under 30 Hz, 1.9 g conditions in mice [65]. Raval, Schatz [66] reported a decrease in inflammatory biomarkers and infarct volume, an increase in BDNF concentration, and the promotion of overall functional recovery when 40 Hz WBV was applied twice a day for 30 days to elderly female mice [66]. In addition, WBV reportedly contributes to the improvement of brain function through activation of the forebrain cholinergic system, increased glucose transport across the blood‒brain barrier, expression of immediate early genes, production of proteins related to synaptic plasticity and neurogenesis, and elevated levels of tyrosine hydroxylase (the enzyme responsible for dopamine precursor synthesis) [67]. Notably, while many of the hypothesized mechanisms are supported by preclinical and EEG-based studies, few of the included RCTs directly measured neurophysiological indicators such as BDNF levels, cerebral blood flow, or cortical activity. Integrating such biomarkers in future trials would provide stronger mechanistic support for WBV-induced cognitive changes.

### Limitations

This meta-analysis has several limitations that should be considered. First, the limited number of included studies is an important limitation. Additionally, this meta-analysis included two studies with a crossover design. The number of cognitive function-related outcomes reported in each study varied, resulting in a total of 35 effect sizes. This provides sufficient evidence for quantitative analysis, although limitations remain regarding qualitative diversity and the verification of long-term effects. In particular, studies with a crossover design may influence the interpretation of results depending on the adequacy of carry-over control or the length of the washout period between interventions, and such design-specific factors could introduce uncertainty into the accuracy of effect size estimation. Second, the limited sample sizes of individual studies may have reduced the reliability of the meta-analysis. Small-scale studies are more susceptible to random error and may result in overestimation or underestimation of effect sizes. Furthermore, smaller sample sizes reduce the statistical power to detect significant associations in meta-regression analyses, particularly in WBV studies where intervention parameters (e.g., frequency, amplitude, and posture) are highly heterogeneous.

Third, the explanation for the physiological and neurobiological mechanisms underlying WBV relies primarily on preclinical animal studies and EEG-based research on short-term cognitive changes. Mechanistic investigations into long-term physiological changes in humans, particularly those involving neurotransmitter activity, cerebral blood flow, or neuroplasticity, remain limited. Therefore, while the current meta-analysis demonstrated a cognitive enhancement effect, the underlying physiological pathways through which these effects are mediated remain unclear. Finally, this study’s meta-regression analysis revealed that the intervention dose (total dose) was a significant moderator. However, prolonged, high-frequency vibration exposure may adversely affect cognitive function [24, 68]. These contrasting results highlight the possibility of a nonlinear dose‒response relationship, necessitating further empirical investigation into whether excessive stimulation induces cognitive fatigue or neurophysiological stress. Future studies should include large-scale, long-term follow-up trials, comparative investigations of standardized WBV protocols, and mechanistic studies that explore the link between cognitive domains and physiological indicators to achieve a more precise understanding of the effects of WBV. Moreover, because optimal WBV intervention parameters (e.g., frequency, amplitude, duration) are likely to differ on the basis of age, sex, and cognitive status, further exploration of tailored intervention strategies is warranted.

## Conclusion

This meta-analysis demonstrated that WBV has a small but statistically significant positive effect on cognitive function, with the greatest improvements observed in global cognition and more pronounced benefits in populations with cognitive decline. The total intervention dose was the only significant moderator, indicating that the cumulative amount of stimulation may be more important than the frequency, amplitude, or duration considered separately.

Although the findings were consistent, with low heterogeneity, little evidence of publication bias, and stable results in sensitivity analyses, most included studies were judged to have a high risk of bias. This calls for cautious interpretation of the evidence.

Overall, WBV appears to be a promising, noninvasive, and accessible intervention for cognitive enhancement, particularly for older adults and individuals with physical limitations. Importantly, across all included trials, no serious adverse events were reported, and adherence was generally high, suggesting that WBV is both feasible and safe in diverse populations. Future studies should clarify optimal dosing strategies, examine domain-specific responses, and apply more rigorous study designs to better inform clinical practice.

## Supplementary Information

Below is the link to the electronic supplementary material.

Supplementary file1 (DOCX 953 KB)

## Data Availability

All the data are available in the supplementary materials or upon reasonable request from the corresponding author.

## Glossary

Whole-body vibration (WBV): A low-intensity physical intervention that delivers mechanical vibrations through a vibrating platform to stimulate neuromuscular and sensory systems.
Overall cognitive function: A pooled meta-analytic estimate representing the combined effect across all reported cognitive outcomes (e.g., global cognition, executive function, attention, and memory).
Global cognition: A domain assessed using standardized screening tools such as the MMSE, MoCA, or ACE-III, reflecting a composite index of orientation, memory, attention, language, and other general cognitive abilities.
Executive function: A set of mental processes that enable individuals to plan, focus attention, remember instructions, and manage multiple tasks effectively.
Amplitude (in WBV): The vertical displacement of the vibration platform during oscillation, typically measured in millimeters (mm).
Frequency (in WBV): The number of vibration cycles per second, measured in Hertz (Hz).
Side-alternating platform: A type of WBV device that oscillates on each side of the body alternately, mimicking a see-saw movement.
