# Supplementary material for: Effects of whole-body vibration on cognitive function: a systematic review and meta-analysis
Source: GeroScience. 2025 Sep 30;48(3):5051–72. doi: 10.1007/s11357-025-01914-0 (PMC13356000; doi:10.1007/s11357-025-01914-0)
Supplement: Supplementary file 1 — Supplementary file1 (DOCX 953 KB) [file 11357_2025_1914_MOESM1_ESM.docx]

**The effects of whole-body vibration on cognitive function: A systematic review and meta-analysis**

**Ji-Woo Seok^a^, Jaeuk U. Kim^a, b^, Jung-Dae Kim^a,*^**

^a^ Digital Health Research Division, Korea Institute of Oriental Medicine, 34054 Daejeon,

South Korea

^b^ KM Convergence Science, University of Science and Technology, Daejeon, South Korea

***Journal**

Sports medicine

*** Corresponding Author**

Jung Dae Kim

Digital Health Research Division, Korea Institute of Oriental Medicine, 1672, Yuseong-daero, Yuseong-gu, Daejeon, Republic of Korea, 34054

email: kjd@kiom.re.kr

Tel (office): +82-042-868-9525

Tel (Fax): +82-042-861-5800

Strategies for Searching the Six Databases Used in the Investigation

**Table S1.** PubMed search strategy.

| 1 | (Cognition [Mesh]) | 218,441 |
| --- | --- | --- |
| 2 | (cognitive function[Title/Abstract]) OR (cognition[Title/Abstract]) OR (global cognition[Title/Abstract]) | 339,818 |
| 3 | (memory[Title/Abstract]) OR (attention[Title/Abstract]) OR (executive function[Title/Abstract]) | 1,141,189 |
| 4 | 1 OR 2 | 33,982 |
| 5 | 1 OR 3 | 114,119 |
| 6 | (Whole body vibration training[Title/Abstract]) OR (Whole body vibration exercise[Title/Abstract]) OR (Whole-body vibration[Title/Abstract]) OR (WBV[Title/Abstract]) OR (whole body vibrations[Title/Abstract]) OR (wholebody vibration[Title/Abstract]) OR (whole body vibration[Title/Abstract]) | 3,146 |
| 7 | 4 AND 6 | 57 |
| 8 | 5 AND 6 | 124 |
| 9 | (randomized controlled trial[Publication Type]) OR (clinical trial[Publication Type]) OR (crossover trial[Title/Abstract]) OR (cross-over study[Title/Abstract]) OR (cross-over design[Title/Abstract]) | 103,158 |
| 9 | 7 AND 9 | 18 |
| 10 | 8 AND 9 | 33 |

**Table S2.** Embase search strategy.

| S1 | 'cognition'/exp | 394,349 |
| --- | --- | --- |
| S2 | 'cognitive function':ab,ti OR cognition:ab,ti OR 'global cognition':ab,ti | 3,824 |
| S3 | 'memory':ab,ti OR 'attention':ab,ti OR 'executive function':ab,ti | 65,306 |
| S4 | 'whole body vibration'/exp | 20,394 |
| S5 | 'whole body vibration training':ab,ti OR 'whole body vibration exercise':ab,ti OR 'whole-body vibration':ab,ti OR wbv:ab,ti OR 'whole body vibrations':ab,ti OR 'wholebody vibration':ab,ti | 4,886 |
| S6 | 'randomized trial'/exp OR 'clinical trial'/exp | 136,075 |
| S7 | 'crossover trial':ab,ti OR 'cross-over study':ab,ti OR 'cross-over design':ab,ti | 6,161 |
| S8 | S1 AND S4 AND S6 | 87 |
| S9 | S1 AND S4 AND S7 | 103 |
| S10 | S2 AND S4 AND S6 | 30 |
| S11 | S2 AND S4 AND S7 | 147 |
| S12 | S1 AND S4 AND S6 | 20 |
| S13 | S1 AND S4 AND S7 | 15 |
| S14 | S2 AND S4 AND S6 | 26 |
| S15 | S2 AND S4 AND S7 | 11 |

**Table S3.** Web of Science search strategy.

| #1 | TS = (cognitive function OR cognition OR global cognition OR memory OR attention OR executive function) | 480,858 |
| --- | --- | --- |
| #2 | TS = (whole body vibration training OR whole body vibration exercise OR whole-body vibration OR WBV OR whole body vibrations OR wholebody vibration OR whole body vibration) | 2,655 |
| #3 | TS = (randomized controlled trial OR clinical trial OR crossover trial OR cross-over study OR cross-over design) | 871,784 |
| #4 | #1 AND #2 AND #3 | 39 |

**Table S4.** Cochrane Library search strategy.

| #1 | MeSH descriptor: [Cognition] explode all trees | 90 |
| --- | --- | --- |
| #2 | (cognitive function OR cognition OR global cognition):ti,ab,kw | 2,346 |
| #3 | (memory OR attention OR executive function):ti,ab,kw | 3,472 |
| #4 | #1 OR #2 OR #3 |  |
| #5 | (Whole body vibration training OR Whole body vibration exercise OR Whole-body vibration OR WBV OR whole body vibrations OR wholebody vibration OR whole body vibration):ti,ab,kw | 312 |
| #6 | (randomized controlled trial OR clinical trial OR crossover trial OR cross-over study OR cross-over design):ti,ab,kw | 9,295 |
| #7 | #4 AND #5 | 44 |
| #8 | #6 AND #7 | 44 |

**Table S5.** Scopus search strategy.

| (TITLE-ABS-KEY("cognitive function") OR TITLE-ABS-KEY("cognition") OR TITLE-ABS-KEY("global cognition") OR TITLE-ABS-KEY("memory") OR TITLE-ABS-KEY("attention") OR TITLE-ABS-KEY("executive function")) AND (TITLE-ABS-KEY("Whole body vibration training") OR TITLE-ABS-KEY("Whole body vibration exercise") OR TITLE-ABS-KEY("Whole-body vibration") OR TITLE-ABS-KEY("WBV") OR TITLE-ABS-KEY("whole body vibrations") OR TITLE-ABS-KEY("whole body vibration") OR TITLE-ABS-KEY("whole body vibration")) AND (TITLE-ABS-KEY("randomized controlled trial") OR TITLE-ABS-KEY("clinical trial") OR TITLE-ABS-KEY("crossover trial") OR TITLE-ABS-KEY("cross-over study") OR TITLE-ABS-KEY("cross-over design")) | 72 |
| --- | --- |

**Table S6.** PsycINFO search strategy.

| all((cognitive function OR cognition OR global cognition OR memory OR attention OR executive function))  AND all(("whole body vibration training" OR "whole body vibration exercise" OR "whole-body vibration" OR WBV  OR "whole body vibrations" OR "wholebody vibration" OR "whole body vibration"))  AND all(("randomized controlled trial" OR "clinical trial" OR "crossover trial"  OR "cross-over study" OR "cross-over design")) | 6 |
| --- | --- |


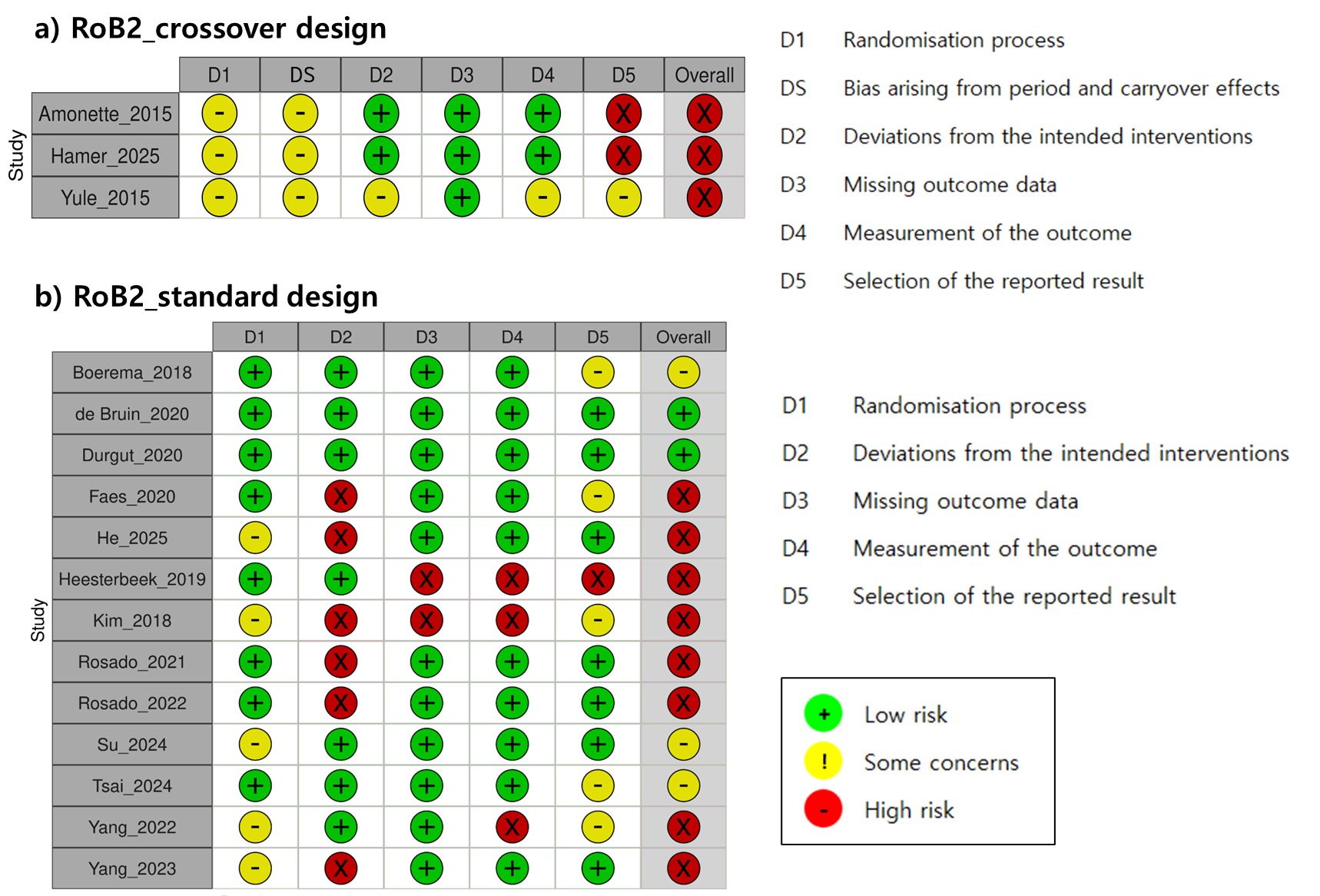


**Figure S1.** Methodological quality assessment of the included studies with Cochrane scale.


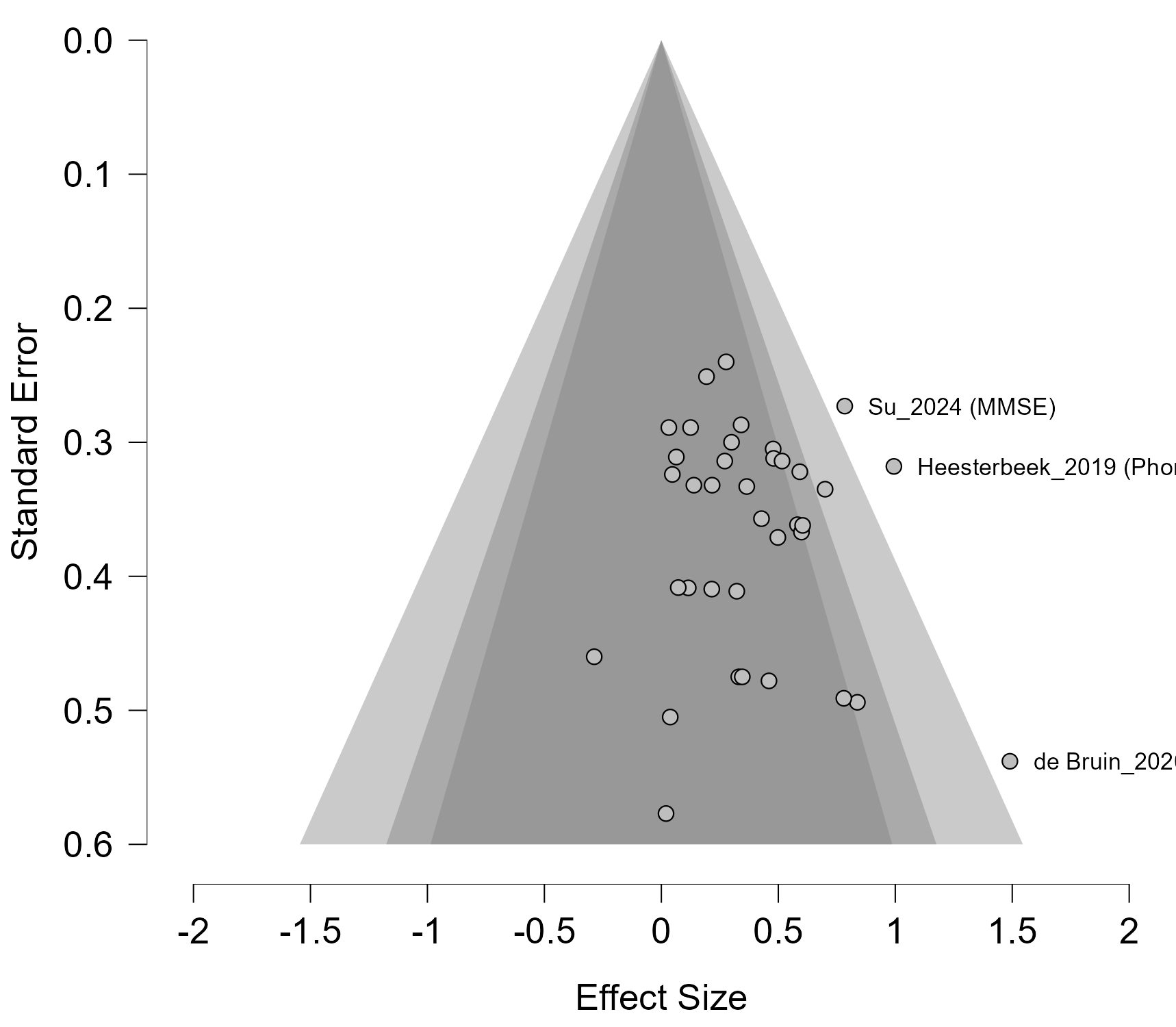


**Figure S2.** Funnel plot assessing publication bias in the meta-analysis of WBV effects on cognitive function.

**Table S7.** The residual heterogeneity estimates in the meta-analysis of WBV effects on cognitive function.

|  | Estimate | 95% Confidence Interval | |
| --- | --- | --- | --- |
|  |  | Lower | Upper |
| τ² | 0 | 0 | 0.144 |
| τ | 0 | 0 | 0.021 |
| I² (%) | 0 | 0 | 14.640 |
| H² | 1 | 1 | 1.172 |

**Table S8.** The result of influence measures.

| **Label** | **Std. Residual** | **DFFITS** | **Cook's Distance** | **Cov. Ratio** | **τ²_(-i)_** | **Q_E(-i)_** | **Hat** |
| --- | --- | --- | --- | --- | --- | --- | --- |
| Amonette_2015 (Vertical WBV, Verbal Memory in ImPACT) | -0.392 | -0.055 | 0.003 | 1.02 | 0 | 24.64 | 0.019 |
| Amonette_2015 (Rotational WBV, Verbal Memory in ImPACT) | -0.128 | -0.018 | 3.192×10-4 | 1.02 | 0 | 24.78 | 0.019 |
| Amonette_2015 (Vertical WBV, Visual Memory in ImPACT) | -0.641 | -0.09 | 0.008 | 1.02 | 0 | 24.39 | 0.019 |
| Amonette_2015 (Rotational WBV, Visual Memory in ImPACT) | -0.747 | -0.105 | 0.011 | 1.02 | 0 | 24.24 | 0.019 |
| Rosado_2022 (TMT-A) | 0.152 | 0.024 | 6.002×10-4 | 1.026 | 0 | 24.78 | 0.025 |
| Rosado_2022 (TMT-B) | 0.581 | 0.093 | 0.009 | 1.025 | 0 | 24.46 | 0.025 |
| Boerema_2018 (Digit Span Forward) | -0.482 | -0.084 | 0.007 | 1.03 | 0 | 24.57 | 0.029 |
| Boerema_2018 (Digit Span Backward) | -0.72 | -0.125 | 0.016 | 1.03 | 0 | 24.28 | 0.029 |
| Boerema_2018 (TMT-B) | -0.029 | -0.005 | 2.529×10-5 | 1.03 | 0 | 24.8 | 0.029 |
| Boerema_2018 (The Stroop color word Test) | 0.986 | 0.17 | 0.029 | 1.03 | 0 | 23.83 | 0.029 |
| de Bruin_2020 (TMT-B) | 2.085 | 0.222 | 0.049 | 1.011 | 0 | 20.45 | 0.011 |
| de Bruin_2020 (TMT-A) | -0.671 | -0.076 | 0.006 | 1.013 | 0 | 24.35 | 0.013 |
| Durgut_2020 (Stroop TBAG) | 0.337 | 0.052 | 0.003 | 1.024 | 0 | 24.68 | 0.024 |
| Durgut_2020 (BRIEF-A Global Executive Composite) | 0.619 | 0.097 | 0.009 | 1.025 | 0 | 24.42 | 0.024 |
| Faes_2020 (Stroop Color Word Test) | -0.418 | -0.102 | 0.01 | 1.06 | 0 | 24.62 | 0.056 |
| Hamer_2025 (Color-Block Test) | -0.881 | -0.177 | 0.031 | 1.04 | 0 | 24.02 | 0.039 |
| Hamer_2025 (Stroop CWIT) | -1.209 | -0.243 | 0.059 | 1.04 | 0 | 23.34 | 0.039 |
| Heesterbeek_2019 (Digit Span Forward) | 0.345 | 0.066 | 0.004 | 1.036 | 0 | 24.68 | 0.035 |
| Heesterbeek_2019 (Stroop word) | 0.344 | 0.064 | 0.004 | 1.034 | 0 | 24.68 | 0.033 |
| Heesterbeek_2019 (Stroop colour) | -1.016 | -0.189 | 0.036 | 1.035 | 0 | 23.77 | 0.033 |
| Heesterbeek_2019 (Stroop interference) | -0.335 | -0.062 | 0.004 | 1.034 | 0 | 24.69 | 0.033 |
| Heesterbeek_2019 (Phonemic fluency) | 1.98 | 0.36 | 0.13 | 1.033 | 0 | 20.88 | 0.032 |
| Heesterbeek_2019 (Semantic fluency) | 0.686 | 0.123 | 0.015 | 1.032 | 0 | 24.33 | 0.031 |
| Heesterbeek_2019 (Digit Span Backward) | -1.027 | -0.183 | 0.034 | 1.032 | 0 | 23.74 | 0.031 |
| Heesterbeek_2019 (MMSE) | -0.119 | -0.024 | 5.807×10-4 | 1.041 | 0 | 24.78 | 0.039 |
| Heesterbeek_2019 (TMT-A) | -1.449 | -0.181 | 0.033 | 1.016 | 0 | 22.7 | 0.015 |
| Kim_2018 (MMSE) | 0.945 | 0.11 | 0.012 | 1.013 | 0 | 23.91 | 0.013 |
| Rosado_2021 (CogTUG) | 0.642 | 0.102 | 0.01 | 1.025 | 0 | 24.39 | 0.025 |
| Su_2024 (MMSE) | 1.534 | 0.327 | 0.107 | 1.045 | 0 | 22.45 | 0.043 |
| Tsai_2024 (Modified Delayed-Matching-to-Sample Task) | -0.253 | -0.049 | 0.002 | 1.037 | 0 | 24.73 | 0.036 |
| Yang_2022 (PASAT-3) | -0.092 | -0.011 | 1.239×10-4 | 1.015 | 0 | 24.79 | 0.014 |
| Yang_2022 (Selective Reminding Test) | 0.831 | 0.097 | 0.009 | 1.014 | 0 | 24.11 | 0.013 |
| Yang_2022 (BRIEF-A Global Executive Composite) | -0.06 | -0.007 | 5.322×10-5 | 1.015 | 0 | 24.79 | 0.014 |
| Yang_2022 (BRIEF-A Metacognition Index) | 0.18 | 0.022 | 4.663×10-4 | 1.014 | 0 | 24.77 | 0.014 |
| Yang_2023 (MMSE) | 0.458 | 0.084 | 0.007 | 1.034 | 0 | 24.59 | 0.033 |
| He_2025 (DSST) | -0.742 | -0.173 | 0.03 | 1.054 | 0 | 24.25 | 0.051 |
| Yule_2015 (ACE-III) | -0.617 | -0.061 | 0.004 | 1.01 | 0 | 24.42 | 0.01 |


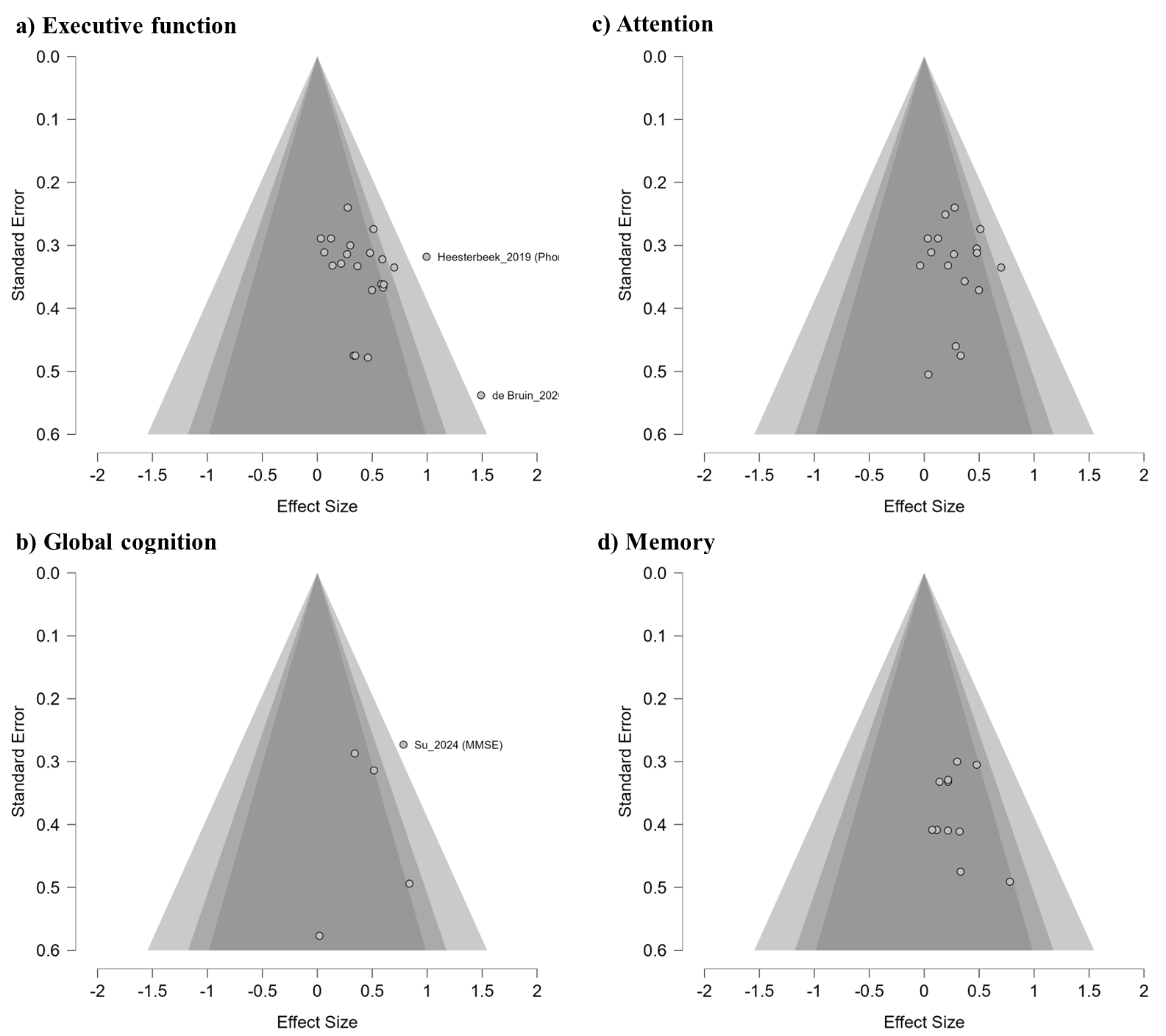


**Figure S3.** Funnel plots for the assessment of publication bias in four cognitive domains.
Each panel presents a funnel plot of standard error against effect size for studies evaluating (A) executive function, (B) attention, (C) global cognition, and (D) memory.

**Table S9.** GRADE assessment of the certainty of evidence for WBV intervention effects on cognitive outcomes.

**Question:** Whole body vibration compared to control condition for individual with or without cognitive impairment?

**Bibliography:**

| **Certainty assessment** | | | | | | | **№ of patients** | | **Effect** | | **Certainty** | **Importance** |
| --- | --- | --- | --- | --- | --- | --- | --- | --- | --- | --- | --- | --- |
| **№ of studies** | **Study design** | **Risk of bias** | **Inconsistency** | **Indirectness** | **Imprecision** | **Other considerations** | **Whole body vibration** | **sham or placebo or usual routine or active control** | **Relative (95% CI)** | **Absolute (95% CI)** |  |  |
| **cognition (follow-up: mean 6.59 weeks; assessed with: Tools assessing global cognition, memory, attention, and executive function)** | | | | | | | | | | | | |
| 37 | randomised trials | serious^a^ | not serious | not serious | not serious | none | 611 | 611 | - | **0**  (0 to 0 ) | ⨁⨁⨁◯ Moderate^a^ |  |
| **Global cognition (follow-up: mean 7.60 weeks; assessed with: MMSE & ACE-III)** | | | | | | | | | | | | |
| 5 | randomised trials | very serious^b^ | not serious | not serious | serious^b^ | none | 93 | 87 | - | **0**  (0 to 0 ) | ⨁◯◯◯ Very low^b,c^ |  |
| **Executive function (follow-up: mean 6.64 weeks; assessed with: Digit Span (backward), Stroop (color-word/interference/CWIT/TBAG), TMT-B, BRIEF (MI), CogTUG, Color-Block Test, Phonemic fluency, Semantic fluency)** | | | | | | | | | | | | |
| 22 | randomised trials | very serious^d^ | not serious | not serious | not serious | none | 425 | 398 | - | **0**  (0 to 0 ) | ⨁⨁◯◯ Low^d^ |  |
| **Memory (follow-up: mean 3.55 weeks; assessed with: Verbal and Visual Memory in ImPACT, Digit Span (forward and backward), PASAT-3, Selective Reminding Test, Modified Delayed-Matching-to-Sample Task, and Semantic Fluency)** | | | | | | | | | | | | |
| 11 | randomised trials | serious^e^ | not serious | not serious | not serious | none | 172 | 162 | - | **0**  (0 to 0 ) | ⨁⨁⨁◯ Moderate^e^ |  |
| **Attention (follow-up: mean 5.47 weeks; assessed with: Digit Span Forward, TMT-A, PASAT-3, the Modified Delayed-Matching-to-Sample Task, and multiple versions of the Stroop Test)** | | | | | | | | | | | | |
| 17 | randomised trials | serious^f^ | not serious | not serious | not serious | none | 354 | 329 | - | **0**  (0 to 0 ) | ⨁⨁⨁◯ Moderate^f^ |  |

**CI:** confidence interval; **SMD:** standardised mean difference

#### Explanations

a. Four out of sixteen included studies were rated as having a high risk of bias overall, particularly due to concerns in the selection of the reported result and missing outcome data. The remaining studies were judged as either low risk or with some concerns. Therefore, the overall risk of bias was rated as serious.

b. Risk of bias was rated as very serious because 5 out of 6 included studies were judged to be at high risk of bias, and the remaining study had some concerns. Common issues included lack of blinding, inadequate allocation concealment, and incomplete outcome data. These substantial methodological limitations significantly reduce confidence in the estimated effect size.

c. Imprecision was rated as serious because the number of included studies was small (n = 6), and the 95% confidence interval for the effect size (SMD 0.538; 95% CI: 0.269 to 0.808) was relatively wide, spanning from a small to a moderate effect. This introduces uncertainty regarding the true magnitude of the effect.

d. Risk of bias was rated as very serious because the majority of the included studies (11 out of 18) were judged to be at high risk of bias, and only a small proportion were at low risk (4 out of 18). Common concerns included lack of blinding, inadequate allocation concealment, and incomplete outcome data, all of which substantially reduce confidence in the estimated effect size.

e. Among the 12 included studies, 9 were rated as having a high risk of bias and 3 as having some concerns, indicating a generally high level of methodological bias. Notably, issues were frequently observed in randomization procedures and outcome measurement.

f. Of the 14 included studies, 8 were rated as high risk of bias and 3 as having some concerns. The primary sources of bias were related to inadequate randomization procedures and the use of unblinded, subjective outcome assessment
